# Univariate associations between housing, management, and facility design factors and the prevalence of lameness lesions in fourteen small-scale dairy farms in Northeastern Algeria

**DOI:** 10.14202/vetworld.2020.570-578

**Published:** 2020-03-27

**Authors:** Zoubida Dendani-Chadi, Khelaf Saidani, Loubna Dib, Fayçal Zeroual, Faouzi Sammar, Ahmed Benakhla

**Affiliations:** 1 Department of Veterinary Medicine, Faculty of Natural Science and Life, University of Chadli Bendjedid, P.O. Box 73 El Tarf, 36000, Algeria; 2 Department of Veterinary Medicine, Saad Dahlab University P.O. Box 270 Blida, 09000, Algeria; 3 Department of Agronomy, Faculty of Natural Science and Life, University of Chadli Bendjedid, P.O. Box 73 El Tarf, 36000, Algeria

**Keywords:** Algeria, housing, lame cows, lesions, management, pasture

## Abstract

**Background and Aim:**

This cross-sectional study aimed to analyze the associations between different types of housing, management, and facilities on the prevalence of lame, causing lesions in smallholder dairy farms in Algeria.

**Materials and Methods:**

The on-site investigation took place between December 2012 and May 2015. All cows were locomotion scored on a four-point scale, and foot lesions causing lame were diagnosed and recorded. Factors related to the farm and the cows’ conditions were also assessed. The association between the possible risk factors and lame lesions was assessed using univariate analysis.

**Results:**

Of the 349 cows evaluated, 13% were lame (lameness score ≥2), with higher lameness values recorded for the hind feet than for the forefeet. Cows without lameness were classified as healthy. The two most frequent lesion diagnoses observed in lame cows were interdigital dermatitis/heel horn erosion (ID/HE; 39%) and interdigital phlegmon (IP; 35%), followed by traumatic lesions (T; 11%), digital dermatitis (DD; 8.7%), and laminitis-related diseases (L; 6.5%). The risk of being lame was increased in large herds with cows of the Holstein breed, and those in the third parity and above. Tie housing, concrete floor, concentrate feeding, zero-grazing, and the use of foot trimming occasionally were associated with increased risk for the presence of lame lesions. The region and footbathing frequency had no association with the prevalence of lame lesions (p≥0.05).

**Conclusion:**

These results have important implications; they indicate that several aspects of housing, management, and facility design are common protective factors for the prevalence of lame lesions. These factors should be maintained correctly to not only reduce the number of lame cows in these herds but also decrease the direct and indirect costs associated with cases of lameness.

## Introduction

In smallholder dairy systems, which dominate our agriculture, Algerian dairy cattle population is estimated at 2 million, with estimated milk production of 3 billion liters [1]. Milk production systems can be broadly categorized into urban, peri-urban and rural, and are located in the north of the country. This area accounts for most of the dairy cow population (60%), forage area (60.9%), and domestic raw milk production (63%). The rest, 26%, occupies the regions with agropastoral vocations and semi-arid and arid climate, and 14% are located in the Saharan region with desert climate [2]. The Brown Atlas is the dominant indigenous breeds in the Northeast of Algeria; reared under the traditional extensive system, and characterized by limited productivity (<1000 kg/cow and per lactation) [1]. Their feeding depends largely on unimproved natural pasture, with seasonal supplementation [3]. Imported breeds mainly black and white Friesians, Holstein, and Montbéliard, and various crosses are reared either under an intensive or semi-intensive system. They produce <4000 kg/cow and per lactation on average, and are mainly concentrated in areas, generally with high irrigation potential, around urban agglomerations [1]. The herd sizes kept under these systems vary between 10 and 20 cows in extensive, 50 cows on average in the intensive and semi-intensive system. The semi-intensive system is practiced in areas of greater land availability and keeps cows on pasture during spring, but pasture is not the main source of feed; cows are housed indoors for a large proportion of the day, receiving silage, and concentrate supplementation. Intensive farming system is essentially “landless,” since the animals are housed and fed. Farmers bring and provide water and chopped or cut grass to their cattle and animals are milked in automatic milking parlors.

The need to increase milk production in Algeria is a necessity, as the country depends largely on imports to fulfill the domestic demand for milk and dairy products. According to Kaouche-Adjlane *et al*. [1], 60% of milk requirements are imported, which costs approximately 769 million dollars/year. Such options have had important consequences on the whole organization of the dairy chains, and on the development of local production and collection of raw milk [1,3]. This inadequate cattle productivity is constrained by several factors such as problems of adaptation of imported breeds, low genetic potential of native livestock, water stress, lack of fodder production, costs of commercial feed, poor access to breeding, and poor herd-health management practice [1,3]. All these factors predisposed the cows to diseases and other stressful conditions. Lameness in cattle is one of the main causes of poor economic output [4-6]. It is the third most important cause of economic loss after reproduction and mastitis [7], and one of the most serious threats to the well-being of dairy cows [8], because it is associated with painful conditions in the locomotory apparatus [7,9,10]. It is a clinical sign with a multifactorial etiology involving infectious, noninfectious, and traumatic injuries [11-13]. Several studies have reported associations between lameness and factors at the herd level such as aspects of housing and flooring [9,14,15], management [16], food and environmental conditions [17], access to pasture, and footbath frequency [18], as well as individual factors such as breed [9,15], parity, and stage of lactation [19], and body condition [8]. These variables contribute to the variation in the lameness of dairy cows [20]. The prevalence of lameness has been assessed in the previous studies and is considered unacceptably high; moreover, the range of lesions that appear is wide and diverse [21]. It is, therefore, crucial for dairy farmers, for the sake of animal health and economy, to control the condition of the feet of their entire herd. The implementation of control measures requires knowledge of the prevalence of lameness and its associated risk factors. Its evaluation is useful also to veterinarians, researchers, and those involved in animal welfare verification programs in their efforts to reduce lameness [22]. To estimate this prevalence, visual observation of gait and posture abnormalities remains the most common method that facilitates earlier identification, quantification, and treatment of lameness lesions [16,22,23].

Despite the above-mentioned research in many countries all over the world, the lameness of Algerian dairy cows has not been sufficiently researched. Although its prevalence has so far been reported by only one other recently published study [24], specific information about risk factors for lameness in Algeria has never been researched before. Thus, our aim was to investigate the risk factors related to environmental conditions, housing, management, and facility design associated with lameness lesions in dairy cows, raised in small Algerian farms, in the northeastern region of the country.

## Materials and Methods

### Ethical approval

This study does not require the approval of the Animal Ethics Committee of the University of Chadli Bendjedid El Tarf, Algeria. No procedures performed in the study affected the animals.

### Study areas

The study was carried out on 14 farms in three provinces of the far Northeast of Algeria. Annaba and El-Tarf (areas I and II) are located on the coastal strip and are subject to a Mediterranean climate, experiencing mild and humid weather in winter and hot and dry weather in summer. The average annual rainfall ranges between 400 and 1000 mm, and the average annual temperature is 18°C [25,26]. At an altitude of approximately 290 m, the Guelma region (area III) is located in the interior of the country and is subject to a harsh climate that alternates between cold and wet winters to hot and dry summers. The annual average rainfall and temperature is 400-500 mm in the south to nearly 1000 mm/year in the north, and 17°C, respectively [26].

### Study design and population

This cross-sectional study included 349 cows from 14 small-scale farms and located in three regions: One farm in region I (Annaba), 11 in region II (El-Tarf), and two in region III (Guelma). A list of farms was previously provided by the Algerian Directorate of Agricultural Services and Fisheries Assistance. All animals of each selected farm were included in the study. Farmers and their veterinarians agreed to participate. No farms were excluded from the study, but animals were excluded from the study for some analyses when data were not available. The cows were managed in two groups: The Holstein cows’ group (highly concentrated feed) and Brown Atlas cows’ group (low concentrated feed and free community pastures outside all year round). One farm (n=65 Holsteins) followed a zero-grazing feeding system (intensive), three farms (n=128 Holsteins) grazed their cows in spring (semi-intensive), and ten farms (n=156 Brown Atlas cows) grazed their cows at free communal pastures all year round (extensive). The farms were classified into two categories: Small (≤30) and large (>30 cows). Nine farms (216 cows) were housed in tie stalls (TS) and five (133 cows) were housed in free stalls (FS). Of the nine TS farms, three (housing 142 Holsteins) had concrete grating flooring with straw bedding, and six (housing 74 Brown Atlas cows) used wood chips bedding over the dirt floor. With regard to the five FS farms, four (housing 82 Brown Atlas cows) used wood chips bedding over the dirt floor while one (housing 51 Holsteins) used sand as stall bases. Cows were also grouped based on the first parity, second parity, and third parity and above. With regard to claw health management practices, four farms had a claw trimming program and three used a footbath (Table-1).

**Table-1:** Distribution of farm and cow-level factors between cows with lame lesions among 14 Algerian small-scale dairy farms and 349 cows included in the analysis (2012-2015).

Risk factors	All cows (n=349)	Farms (n=14)	Lame cows

			Number (%)
Region			
Annaba (I)	51	1	8 (15.6)
El Tarf (II)	221	11	25 (11.3)
Guelma (III)	77	2	13(16.8)
Breed			
Holstein	193	4	44 (22.8)
Brown of atlas	156	10	2 (1.3)
Herd size (number of animals)			
≥10	29	3	0 (0.00)
11-30	141	8	4 (2.8)
>30	179	3	42 (23.4)
Season			
Winter	349	14	34 (74)
Spring	349	14	12 (26)
Housing system			
Tie-stalls	216	9	38 (17.6)
Free-stalls	133	5	8 (6.01)
Flooring system			
Solid concrete	142	3	36 (25.35)
Earthen floor	156	10	2 (1.3)
Sand	51	1	8 (15.7)
Feeding			
Highly concentrated feed	193	4	44 (22.8)
Low concentrated feed	156	10	2 (1.3)
Grazing			
Grazing period	128	3	21 (16.4)
Zero-grazing	65	1	23 (35.4)
Pasture all years	156	10	2 (1.3)
Parity			
1^st^ parity	76	14	3 (3.9)
2^nd^ parity	82	14	12 (14.6)
≥3^rd^ parity	191	14	31 (16.2)
Presence of a footbath			
Yes	128	3	21 (16.4)
No	221	11	25 (11.31)
Periodical foot trimming			
Once a year	128	3	21 (16.4)
Occasionally	65	1	23 (35.4)
Not at all	156	10	2 (1.3)
Lame lesions			
ID/HE	18 (39)	3	18 (39)
IP	16 (35)	4	16 (35)
DD	4 (8.7)	2	4 (8.7)
L	3 (6.5)	2	3 (6.5)
T	5 (11)	3	5 (11)
All diagnoses	46 (13.2)		46 (13.2)
Limb affected			
Fore limb	11 (24)		11 (24)
Hind limb	35 (76)		35 (76)

ID/HE=Interdigital dermatitis/heel horn erosion, IP=Interdigital phlegmon, DD=Digital dermatitis, L=Laminitis-related diseases, T=Traumatic lesions

### Data collection

Due to practical reasons and availability of students, farms were visited from December 2012 to May 2013 in region I, December 2013 to May 2014 in region II, and December 2014 to May 2015 in region III. Each farm from each region was visited twice monthly for 6 months. All data were collected by the same two trained observers from the University of Chadli Bendjedid, Department of Veterinary Medicine, in El-Tarf. They are helped by two students during this visit. Farmers received a questionnaire to be completed the same day. Using farm records, the following data were collected for each cow and farm during initial farm visits: Calving date, herd size, cattle breed, age of livestock, housing, flooring, management, nutrition, cleanliness of the herd, and frequency of footbath, and claw trimming. During the monitoring visits, all changes in the condition of the cattle and their environment after the first visit were recorded. The effect of milk production was not taken into account in this study, as precise information on monthly milk production was not available for Brown Atlas cows.

### Diagnosis of lameness lesions

To minimize the potential for between observer bias, all assessors (n=4) underwent a training session for lameness scoring before the start of the study, and assessment of locomotion, by an experienced locomotion scorer. Cases of lameness were observed and recorded by farm staff and confirmed by the veterinarian of the same herd and the two authors. We chose the DairyCo mobility score, suggested by Barker *et al*.[27], for its ease of use and to allow for rapid training of assessors, as the rating is based on animal locomotion alone, and is designed for practical use on the farm. The same two trained observers performed the locomotion scoring on all farms, using a four-point scoring scale, where 0=sound locomotion, 1=imperfect locomotion, 2=lame, and 3=severely lame, as shown in Table-2. Holstein cows were observed when they leave the milking parlor or an outdoor exercise area. In the case of Brown Atlas cows, scores were obtained by observing them in the pasture. Cows were defined as lame if limping was present, when walking, which was equivalent to a score of ≥2 on at least one of all visits. Cows without lameness were classified as healthy. At trimming, foot disorders in the lame cows were diagnosed, and they were treated in accordance with the “Dutch trimming method” [28]. The reports on foot disorders were written jointly by the veterinary practitioner and the two authors. We used a reference sheet with illustrations and descriptions of the different levels of foot lesions to assist in the recognition of lesions according to the Merck veterinary manual [29]. Only the presence or absence of lesions, without the notion of severity or gravity, was taken into account. Depending on the location of the different claw areas described by Greenough and Vermunt [30], two categories of lesions were recorded: Lameness lesions in the skin and interdigital space and in-claw lesions. Heel horn erosion (HE), which is considered a secondary complication to interdigital dermatitis (ID) [31-33], interdigital phlegmon (IP), and digital dermatitis (DD) are the most common infectious lesions of the skin and interdigital space [34]. Laminitis-related lesions are the most common non-infectious lesions of the claw. Hoof overgrowth, subacute laminitis, or chronic laminitis were coded “L.” Stones and foreign bodies embedded in the foot as well as an injury in the sole and interdigital space were coded as traumas “T” [11].

**Table-2:** Lameness scoring scale dairy Co-adapted by Barker *et al*. [27].

Mobility score	Criteria
0	Sound/Perfect gait
1	Abnormal locomotion, but not favoring any particular limb/tender-footed gait
2	Lame and uneven or arched back
3	Severely lame with score “2” conditions and a very slow gait. Slower than the pace of “brisk walk” by a human

### Statistical analysis

The prevalence of lame lesions was computed by dividing the total number of cows observed with a locomotion score ≥2 by the total number of cows. Pearson’s Chi-square test was used to compare the prevalence of lameness in the univariable analysis. An association was considered significant at the level of p<0.05. The statistical analyses of data were performed using R Version 3.3.3. [35].

## Results

### Prevalence of lame lesions

The descriptive results of the prevalence of lame lesions and associated risk factors are summarized in Tables-1 and 3. The overall prevalence of lame lesions was 13% (lameness score ≥2), across the 14 farms, and the within-regions prevalence scores were 15.6%, 11.3%, and 17% in Annaba, El-Tarf, and Guelma, respectively. In the cows, 24% of lameness lesions were in the forefeet and 76% were in the hind feet. All affected cows were lame only in one foot. Infectious lameness lesions, including ID/HE, IP, and DD, were recorded in 82.6% of lame cows, and non-infectious lameness lesions, including L and T, were recorded in 17.4% of lame cows. This difference was statistically significant (p=0.002). ID/HE and IP were the most frequently diagnosed diseases as causes of lameness, accounting for 39% and 34.9% of the total, respectively, followed by T (11%), DD (8.7%), and L (6.5%). Statistically significant differences were observed between the prevalence rates of lame lesions (p<0.001).

**Table-3:** The variables tested in the univariable analysis for association with lame lesions in 14 Algerian small-scale dairy farms and 349 cows (2012-2015).

Risk factors	χ^2^^a^	df	p-value
Region			
Annaba (I)	1.87643	2	p=0.39
El Tarf (II)			
Guelma (III)			
Breed			
Holstein	17.7144	1	p<0.001
Brown of atlas			
Herd size (number of animals)			
≤10	41.7798	2	p<0.001
11-30			
>30			
Husbandry system			
Intensive	34.9000	1	p<0.001
Extensive			
Season			
Winter	11.6419	2	p=0.003
Spring			
Housing system			
Tie stalls	9.64193	1	p=0.002
Free-stalls			
Flooring system			
Solid concrete	37.9636	2	p<0.001
Earth floor			
Sand			
Feeding			
Highly concentrated feed	34.9000	1	P<0.001
Low concentrated feed			
Parity			
1^st^ parity	22.7839	2	p<0.001
2^nd^ parity			
≥3^rd^ parity			
Periodical trimming			
Once a year	48.4685	2	p<0.001
Occasionally			
Not at all			
Footbaths			
Yes	1.83802	1	p=0.18
No			
Lameness			
HH/ID	22.478	4	p<0.001
IP			
DD			
L			
T			
All diagnoses			
Foot lame lesions			
IFL: Infectious foot lesions	10.9466	1	p=0.002
NIFL: Non-infectious foot lesions			

a Chi-square Pearson’s test, df=Degrees of freedom, IFL=Infectious foot lesions, NIFL=Non-infectious foot lesions

### Risk factors analysis

The percentage of lame lesions at the cow and farm-level factors is reported in Table-1 and its significance in Table-3. Two individual factors increased the risk of lame: The Holstein cows (22.8 vs. 1.3% for Brown Atlas cows), and third parity or above (16.2 vs. 3.9% in 1^st^ parity). At the farm level, the prevalence of lame lesions was significantly associated with cows reared under zero-grazing (35.4 %) compared to grazing period (16.4 %) and pasture all year (1.3%), and cows kept in tie housing (17.6%) compared to free-housing (6.01%), and those with concrete floor (25.3%) in comparison to sand (15.7%) or dirt stall bases (1.3%), winter housing (74 vs. 26% in spring), and concentrate feeding (22.8 vs. 1.3% for low concentrated feed). In addition, the risk for the lame lesions was higher in large herds >30 cows and those that used a foot trimming occasionally (35.4%) compared to those that did regularly (16.4%). However, on pasture all year, the lowest rate is recorded when cows have not been trimmed at all (1.3%). Non-significant (p≥0.05) association between the prevalence of lame lesions and the region and footbath was observed.

## Discussion

Few published articles report on the prevalence of epidemiological lameness cows in regions outside the United States, the Netherlands, and the European countries [36]. A noted strength of our work is that this is the first published study which has provided information on the environmental, management, and facility practices risk factors associated with lame lesions in Algerian dairy cattle. This data should provide a reference point for comparison with data from other countries. We and managers have more opportunities to observe each individual cow as she walks in order to detect lameness because most of the smallholder dairy cattle are family managed and are easy to manage due to proximity of the animals. However, several negative factors for animal welfare and milk production are mentioned earlier [3], cattle diseases are also common, especially the high prevalence of parasitic, including tick-borne diseases [37], particularly in herds that graze, whose exposure to ticks is greater. Anaplasmosis and piroplasmosis are also prevalent, especially in low land humid areas [38]. We also encountered several problems associated with the collection and access to registered data on past farming practices, herd management, and performance that is lacking for most extensive farms. The presence of other livestock species, such as dogs, sheep, and poultry is also frequent.

Several limitations exist in this study that should be addressed with future research. First, our data come from small herds; hence, this study population cannot be considered representative of Algerian dairy herds in general. Second, lameness cannot necessarily be identified and recorded in a comparable way [6,23,39]. Third, several specific combinations of risk factors have been associated with an increased prevalence of lame cows, and it has been difficult to conduct a comparison with the results of other studies and countries [9,23]. In addition, the previous research has shown that inter-rater reliability for locomotion scoring can be variable [6]. Here, interobserver reliability for lameness scoring was not assessed. However, we believe that the implemented training session, the same two trained observers, the simplest gait score, and his frequent use, the small herd size, and periodic visits throughout the study; collectively, these contributed to the achievement of high interobserver agreement. The effect of milk production and the weight of the cow that were not taken into account in this study, as precise information on monthly milk production, were not available for Brown Atlas cows, as detailed and representative information are difficult to collect. Hence, these factors might explain these differences in risk of lameness between Holstein and the Brown Atlas cows.

Data on the overall prevalence of lameness (13%) support the only Algerian study (12.7%) [24] and are consistent with those presented in related studies from Kenya (11.7%) [40], Spain (13.8%)[41], Switzerland (14.8%) [42], Australia (18.9%) [17], some other European countries (18%) [39], and Malaysia (19%)[43]. However, the estimate of lameness prevalence in this study was lower than that of farms in the Northeastern United States (55%) [44], England and Wales (37%) [27], Canada (36%) [13], Brazil (35%) [45], Austrian and German (34%) [9], and China (31%) [18]. Accordingly, Green *et al*. [46] indicate that the prevalence of lameness in dairy cows is high in developed countries. However, recent studies in North America have reported a lower prevalence of 13.2% [47] and 7.2% [14]. This high variability in lameness estimates is reported worldwide. Some of the variations can be attributed to the difficulty in defining clinical lameness in dairy cows [5], as well as the difference in lameness scoring system used, and interobserver reliability for lameness scoring [6]. Part of the variation may also be attributed to the different skills of personnel responsible for identifying lame cows, in sample size, climate, and disease status of infectious claw diseases, breed selection, and productivity of the cows [15,46]. On the other hand, infectious causes of lameness were predominant in this study, accounting for 82.6% of all diagnoses. Among the lame infectious lesions recorded, ID/HE and IP were the most common causes of lameness, DD was reported less commonly. Somers *et al*. [34], Frankena *et al*. [48], and Refaai *et al*. [33] obtained similar results for associations between lameness and infectious lesions such as ID/HE and DD in their studies. Possible reasons for the high prevalence of infectious lesions are the unhygienic environmental conditions, such as the accumulation of manure, feces, and urine, which are considered important predisposing factors [34]. These diseases are highly transmissible, especially when floors are not properly cleaned [49], as was the case in this study.

Several risk factors were associated with the prevalence of lame in this study. In terms of the breed effect, Holstein cows had the highest risk of lame lesions than did Brown Atlas cows. Our results are consistent with several studies comparing jersey [20,22,45], crossbreeds [27], Ayrshire [15], and other breeds [17,39] to Holstein cows. The reason for this could be that lameness has been indicated as a major problem in Holstein herds [27]. In addition, Holsteins are on average bigger and have higher milk yield, which can be associated with more concentrated feeding [15] that, in turn, could predispose them directly or indirectly to lameness problems [20,50]. On the other hand, the composition of breeds varied considerably from country to country, with some breeds being very country-specific [39]. The Brown Atlas is a light and thin cow, permanently housed in pastures and fed a low-carbohydrate, and high-fiber diet.

Lame lesions prevalence increased with increasing parity. This is partly in agreement with several studies which found more lameness with increasing age [9,45]. This association may be explained by the fact that older cows are predisposed to relapse with certain foot lesions and are exposed for longer intervals to the housing environment than are younger cows [6,15].

The prevalence of lameness, and the lesions associated with the lameness, varies widely between different management systems; this prevalence is high in the farms of the intensive and semi-intensive system, in which cows produced more milk, received a large proportion of their feed as silage and grains, and spent a large proportion of the day indoors [51]. From experiences in practice, it has been suggested that the prevalence of lameness is higher when cattle stay indoors all year round [17,40,50]. However, the lower levels of lameness in an extensive farming system may have been a result of the high use of pasture and the use of genotypes adapted to the environment, and low levels of supplementation and lower milk production; similar results have been observed elsewhere [42,51]. Access to pasture is known to be beneficial for foot health; it reduces the risk of lameness by providing a large housing surface, beneficial claw wear, good traction, and low levels of fecal bacteria [39,52]. The risk factors for lameness in the pasture are related to the risk of trauma, for example, from long walking distances and lack of track maintenance [51], as was the case in this study.

Several studies [9,14,15] have estimated the prevalence of lameness lesions in dairy cattle and some have shown associations between housing and lameness. It has been frequently reported that FS housing increases the incidence of lameness compared to tie housing [12,13,23]. However, in our sample, we observed a higher prevalence of cows with lame lesions confined in TS. We revealed that FS with sand or dirt bases were associated with a lower prevalence of lameness in cows than were solid flooring TS. This finding can be attributed to the type of stall base, which is more important than the type of housing system. Studies [4,18] found a higher prevalence of lameness in FS than in TS but not when FS with sand as the stall base were compared with non-sand TS. Faye and Lescouret [53] reported fewer cases of lameness in farms with dirt stall bases than in farms with concrete stall bases, which can be attributed to the fact that the ground is soft, flexible, and nonabrasive, and ultimately reduces the risk of lameness. The use of solid concrete bedding can affect digital health, as continuously wet, irregularly scraped, and insufficiently mulched conditions favor infectious diseases [54], as was the case in this study. The hardness, abrasiveness, and slipperiness of such stall bases have adverse effects on animal welfare and health [12,19].

In general, the larger the herd size, the greater the risk of lameness. In accordance with this observation, some studies [22,55] found that in a large herd, more procedures are mechanized than in a small herd. A high degree of mechanization can reduce the time the farmer spends with each cow, which could be associated with a higher frequency of lameness [20]. Diets with a high concentrate to roughage ratio have traditionally been considered as one of the major risk factors for lameness [19]. This diet is one of the primary underlying causes of laminitis [17], particularly the consumption of high-concentrate diets, which are more common in Holstein than in Brown Atlas cows in this study. Furthermore, dairy cows with high genetic potential for milk yield have been shown to have an increased risk of lameness. Other factors associated with nutrition, such as frequency of feeding and feed composition [39], may be of greater importance in terms of lameness, but these were not investigated.

This study also revealed that farms that trimmed claws of cattle at decreased frequencies (occasionally) had an increased prevalence of lame cows than did those that routinely trimmed claws of cattle. Results from prior studies [34,41,53] have shown that long intervals between claw trimmings are associated with increased lameness. Studies have also revealed that farms where cattle’s claws are trimmed only when they are overgrown or when lameness has occurred have higher levels of lameness [56]. However, with regard to cows raised on pastures, we show that the lowest rate of lameness is recorded when their claws have not been trimmed at all. Here, when cattle graze in pastures all year round and walk long distances every day on gravel or concrete, claw trimming is rarely necessary. Claw trimming has many proved positive effects that aim to restore finger health and improve animal welfare, as other studies report [19,34,43], but it is not the only way to prevent lameness, because possible lameness problems caused by the trimming process have also been reported. It was not possible to assess the quality of claw trimming, and it is likely that incorrect trimming techniques could worsen the problem. In general, poor-quality trimming has been recognized as a factor contributing to the occurrence of locomotion disorders, exposing the feet to all kinds of injuries and pathogen penetration [57]. Excessive hoof wear and tear can cause instability on concrete thus creating pain and discomfort for the cow, leading to a higher probability of recurrence of lame lesions [42].

Footbath was not a general practice in most of the studied farms; only three farms were using the footbath on a regular basis. The routine use of a footbath was associated with a greater prevalence of lameness in cows, as other studies have also reported [11,27,58]. Although, many studies have shown that routine footbathing is associated with a lower prevalence of lameness [55,59]. In this study, only the presence or absence of a footbath is taken into account; the design, protocols of footbaths, and choice of disinfectants vary considerably [31,59]; however, their effects on lameness were not estimated. For disinfectants, copper sulfate, Javel water, and Cresyl solutions (5%) were the most commonly used materials in our footbath systems. These materials are very disadvantageous for the environment [31], as they deteriorate rapidly in the presence of a very high amount of organic matter. This phenomenon makes them unsuitable for use in footbaths. Considering the factors of race, grazing and housing, considering the disinfectants used, it is believed that our results are very much due to improper use of footbath instead. The presence of a foot bath has to be seen as an effect of higher lameness prevalence rather than as a cause. The benefit of the use of footbathing under our conditions is less clear and further research is required.

## Conclusion

Any measures aimed to reduce the prevalence of lameness should be geared toward minimizing the constraints experienced by the smallholder dairy farmers. The identification, treatment, and prevention of lameness are a challenge to dairy farmers, with the causes being multifactorial and involving factors such as nutrition, management practices, housing, and environmental conditions. In confinement, cattle need to be provided with amble comfort by providing good housing and environmental conditions. Deep bedding (sand or dirt bases) provides the best comfort and contributes the least to lameness. For cattle with infectious lesions, control will need to aim at the hygiene of the floors, stalls, and foot. It is essential to provide feeding strategy of cattle living under the extensive system since the pasture forage has lower nutritional value. Farmers in the study area should be encouraged to use locomotion scoring tool to identify lame cows. The benefit of routine claw trimming and the use of footbathing under our conditions are less clear and further research is required.

## Authors’ Contributions

ZD, LD, and FZ participated in the conceptual aspect and design of the study, collected the data, and prepared the manuscript. KS and FS contributed to analyzing data. AB revised and corrected the manuscript. All authors read and approved the final version of the manuscript.
